# A92 MULTIFACETED STRIDE II-BASED MONITORING FOR INFLAMMATORY BOWEL DISEASE ADVANCED THERAPY STARTS

**DOI:** 10.1093/jcag/gwae059.092

**Published:** 2025-02-10

**Authors:** D Parsons, S A MacKay, S Hoque, F Hoentjen, L Dieleman, M Gozdzik, K Kroeker, K Wong, T McMullen, F Peerani, B Halloran

**Affiliations:** University of Alberta Faculty of Medicine & Dentistry, Edmonton, AB, Canada; General Internal Medicine, University of Alberta, Edmonton, AB, Canada; University of Alberta Faculty of Medicine & Dentistry, Edmonton, AB, Canada; University of Alberta Faculty of Medicine & Dentistry, Edmonton, AB, Canada; University of Alberta Faculty of Medicine & Dentistry, Edmonton, AB, Canada; University of Alberta Faculty of Medicine & Dentistry, Edmonton, AB, Canada; University of Alberta Faculty of Medicine & Dentistry, Edmonton, AB, Canada; University of Alberta Faculty of Medicine & Dentistry, Edmonton, AB, Canada; University of Alberta Faculty of Medicine & Dentistry, Edmonton, AB, Canada; University of Alberta Faculty of Medicine & Dentistry, Edmonton, AB, Canada; University of Alberta Faculty of Medicine & Dentistry, Edmonton, AB, Canada

## Abstract

**Background:**

Inflammatory bowel disease (IBD) outreach monitoring has been shown to be cost-effective and reduce healthcare utilization. IBD STRIDE-II guidelines recommend monitoring patient-reported outcomes (PRO’s), biomarkers (e.g., fecal calprotectin (FCP), C-reactive protein (CRP)), and endoscopy to determine if patients achieve defined therapeutic targets. Previous monitoring in the literature has focused on PRO’s alone, which risks undertreating asymptomatic inflammation in spite of elevated flare and colorectal cancer risk. We designed a protocol to closely monitor biomarkers and PRO’s.

**Aims:**

We aimed to assess STRIDE-II based clinical response and facilitate responsive disease management for new advanced therapy start patients.

**Methods:**

Patients complete 24 weeks of outreach monitoring. PRO’s are obtained on days 0, 3, and 7, and every 2 weeks thereafter. Serum labs are collected at baseline and weeks 4, 8, 12, 16, and 24. FCP is collected at baseline and weeks 8, 16, and 24. Medication adherence is assessed at baseline and weeks 12 and 24. Endoscopy is booked 6-12 months from the start date. Treating gastroenterologists receive granular clinical reports summarizing results, the date of last IBD review, and last flare.

**Results:**

75 protocol patients on the following therapies: ustekinumab (n = 31), risankizumab (n = 17), tofacitinib (n = 14), upadacitinib (n = 10), vedolizumab (n = 2), and infliximab (n = 1) were monitored in our protocol so far. 51 patients (68.0%) had failed 1+ prior advanced therapies and 13 (17.3%) had prior IBD surgery. 7 (9.3%) were switched from their initial agent before 24 weeks due to lack of response. 1 (1.3%) had an adverse drug event. 64 patients (85.3%) complied with all FCP collections and 73 (97.3%) completed 95+% of PRO’s. Per STRIDE-II definitions, 47 patients (62.7%) demonstrated clinical response during the protocol and 25 (33.3%) achieved remission by 24 weeks. 15 (20.0%) received steroid courses, 9 (12.0%) presented to an emergency department, and 5 (6.7%) were hospitalized for IBD during monitoring.

**Conclusions:**

Our proactive monitoring protocol provided granular real-world clinical response data to treating physicians. Patients were highly compliant with serial PRO, FCP, and serum labs collection. The protocol facilitates responsive disease management and may lead to treatment-specific monitoring recommendations. In future, we will compare protocol patients to standard of care managed patients to learn if the protocol reduces healthcare utilization and improves patient outcomes and quality of life.

**Funding Agencies:**

None

